# Stressing State Analysis of an Integral Abutment Curved Box-Girder Bridge Model

**DOI:** 10.3390/ma12111841

**Published:** 2019-06-06

**Authors:** Jun Shi, Jiyang Shen, Xiaohui Yu, Junran Liu, Guangchun Zhou, Pengcheng Li

**Affiliations:** 1 School of Transportation Science and Engineering, Harbin Institute of Technology, Harbin 150090, China; hitshijun@hit.edu.cn; 2 Key Lab of Structures Dynamic Behavior and Control of the Ministry of Education, Harbin Institute of Technology, Harbin 150090, China; ranran25805280@163.com (J.L.); gzhou@hit.edu.cn (G.Z.); 17S033081@stu.hit.edu.cn (P.L.); 3 Key Lab of Smart Prevention and Mitigation of Civil Engineering Disasters of the Ministry of Industry and Information Technology, Harbin Institute of Technology, Harbin 150090, China

**Keywords:** stressing state, mutation, updated failure load, stressing state mode, integral abutment curved box-girder bridge

## Abstract

This paper experimentally investigates the working behavior characteristics of an integral abutment curved box-girder (IACBG) bridge model based on the structural stressing state theory. First, the stressing state of the bridge model is represented by generalized strain energy density (GSED) values at each load *F_j_* and characterized by the normalized GSED sum *E_j_*_,norm_. Then, the Mann-Kendall (M-K) criterion is adopted to detect the stressing state mutations of the bridge model from *E_j_*_,norm_-*F_j_* curve in order to achieve the new definition of structural failure load. Correspondingly, the stressing state modes for the bridge model’s sections and internal forces are reached in order to investigate their variation characteristics and the coordinated working behavior around the updated failure load. The unseen knowledge is revealed by studying working behavior characteristics of the bridge model. Therefore, the analytical results could provide a new structural analysis method, which updates the definition of the existing structural failure load and provides a reference for future design of the bridges.

## 1. Introduction

Nowadays, bridge design should not only consider safety, applicability, and aesthetics, but also pay attention to reducing maintenance or even be ‘free maintenance’ in the future, in order to achieve the lowest total price of the whole life of the bridge [1]. In practical use, expansion joints are usually the most vulnerable and difficult to repair for bridges, and their damage could cause various degrees of defects, resulting in corrosion of the structure, expansion failure, etc. [2,3]. Therefore, compared with other types of bridges, integral abutment bridges, which have superior service performance and free maintenance design, have been the first choice of medium and small span bridge and widely applied and researched in the world [4,5]. While relative to the practice of these bridges, the theoretical researches still lag behind leading to the strict limitation of application range, mostly concentrating on straight and oblique bridges for the time being [6,7]. Curved bridges are generally used in highways, urban overpasses, and viaducts due to their small footprint and the ability to meet the requirements of various directions, so the application of integral abutment in small and medium span curved bridges will bring significant economic and social benefits [8,9]. However, the combination of special force characteristics for the two types of bridges, such as bending-torsional coupling effect, soil-structure interaction, etc., must make performance researches more complicated, so many countries or regions still take a conservative and cautious attitude towards the application. Hence, it is necessary for researchers to further study the structural working behavior characteristics of them, so as to provide a theoretical basis for engineering practice. 

As for the study on the mechanical performance of integral abutment bridges, the major research directions consist in soil-structure interaction, thermal effects, and so on. For example, Kerokoski et al. discovered that the cyclic displacement and rotation of abutments could impose extra load on pile foundation, which prominently reduced the vertical bearing capacity of pile foundation [10]. At the same time, other researchers also found that the characters of abutments backfill, the size and pre-drilling filler of piles, etc., had a significant influence on the performance of abutments and bridge girders [7,11,12]. Compared with traditional bridges, the connections among piles, abutment and bridge girder are required to secure sufficient stiffness, rotational stiffness, and bearing strength for integral abutment ones [13], hence, a modified Ramberg-Osgood cyclic model was introduced to obtain the tangent stiffness of the nonlinear spring elements in integral abutment bridges for studying the piling stresses and pile-soil interaction [14]. In addition, due to the removal of expansion joints, parametric study and stress analysis on the thermal response of integral abutment bridges were also researched [15,16]. With regard to curved girders, they suffer the mix of deformation of torsion, bending, and shear resulting in the co-existence of bending normal stress, warping stress, and shear stress in various parts of the structure. When curved girders enter the elastic-plastic stage, their bending and torsional stresses no longer maintain the previous proportion leading to complex structural stressing state [17,18]. Therefore, the introduction of integral abutments seriously raises the difficulties for analyzing the working behavior of curved bridges. For the time being, some researchers have established detailed and three dimensional finite element models of IACBG bridges according to the monitor of practical bridges and evaluate the effects of bridge curvature, skew and abutment backfill soil type under thermal loading [19,20]. However, other researches on bearing capacity, interaction between structure and soil, etc. are quite few, so many research achievements about factors and structural failure mechanisms have not been unanimously approved [21,22]. In addition, the main calculation models and theories for integral abutment bridges, including two-dimensional continuous beam, a two-dimensional frame model with length equaling pile and three-dimension spring-frame, may be inapplicable and too complex [11,23,24]. Generally, due to the uncertainty of abutments backfill and the complexity of structural stress, IACBG bridges only adopt the semi-empirical and semi-theoretical methods to design practical structures and to determine the ultimate bearing capacity of them. As a consequence, it is essential to deeply investigate coordination working behavior and structural failure mechanism for IACBG bridges, so as to establish an accurate and simple stress analysis method. In addition, the high experimental cost makes it difficult to investigate sufficient experiments, which leads to achieving deficient experimental data and failing to improve the existing analytical methods and calculating theories. Yet, the severely limited experimental data still need to apply an effective method in order to analyze the working behavior of IACBG bridges and reveal the hidden knowledge. Hence, it is the key to seek out an innovative method for the sake of discovering unseen knowledge. 

In an attempt to better understand the structural stressing state characteristics of IACBG bridges, authors study the experimental data of an IACBG bridge model based on the theory of structural stressing states [25]. The generalized strain energy density (GSED) sum is adopted to investigate the structural stressing state, and then the structural stressing state mutations are distinguished by an innovative application of the Mann-Kendall (M-K) method. Hence, an updated definition of structural failure and a general criterion for distinguishing the structural failure load are put forward. And the hidden working behavior characteristics of the bridge are also revealed through the innovative method base on structural stressing state theories.

## 2. Structural Stressing State and M-K Criterion 

### 2.1. Structural Stressing State Concept

Under a certain loading case, the mode how the components/units or their combinations (including the whole structure) in a structure behave is defined as structural stressing state by Zhou et al. [26,27,28]. Structural stressing state mode can be stated by the formed matrix or vector of structural response data such as strain, stress, strain energy, displacement (DSPL), and internal force. Structural stressing state is generally for the whole structure and component stressing state for individual structural components, local parts, and internal forces.

Structural stressing state will change/evolve with the load increase and exhibit different characteristics at some special load levels in accordance with the natural law from quantitative change to qualitative change of a system [29]. So when the load reaches a certain value, the structural stressing state certainly will be a qualitative mutation (shape change or magnitude change of stressing state mode), which reveals the inherent and essential property of structures. The authors define the load corresponding to the qualitative mutation of structural stressing state as updated failure load, which differs from the existing definition. 

### 2.2. Numerical Description of Structural Stressing State

The stressing state of a loaded structure is defined as the structural working behavior, characterized by the distribution patterns of strain energy density values, strains, displacements and stresses of key points. Generally, the strain energy density *E*^k^ of the kth point can be calculated by

(1) $$E^{k}=\int \sigma_{1}d\varepsilon_{1}+\sigma_{2}d\varepsilon_{2}+\sigma_{3}d\varepsilon_{3}$$

where *σ*_1_, *σ*_2_, *σ*_3_ and *ε*_1_, *ε*_2_, *ε*_3_ are three principal stresses and strains, respectively. Considering the concept of strain energy density, the generalized strain energy density (GSED) is selected as a characteristic parameter, which expresses the stressing state at a point [30]. Thus, Equation (1) is simplified as,

(2) $$E_{ij}=\frac{1}{2}\sigma_{ij}\varepsilon_{ij}$$

where *E_ij_* is the GSED sum of the *i*th cross section at the *j*th load, *σ_ij_* and *ε_ij_* are respectively the steel bars stress and strain of *i*th cross section at the *j*th load and *σ_ij_* is calculated according to the constitutive equations of the steel.

In order to exclude the influence of units and other factors, the GSED sum value is normalized through Equation (3),

(3) $$E_{j,\mathrm{norm}}=\frac{\sum_{i=1}^{N}E_{ij}}{E_{M}}$$

where *E_j,_*_norm_ is the sum of the normalized GSED values of all the measured points to the *j*th load. *E_M_* is the maximum strain energy value over the loading process. As a scalar, the GSED value of a component/unit is easy to be integrated to characterize the component/unit behavior of the structure. Therefore, the structural stressing state of the whole structure can be appropriately characterized by the sum of the GSED values. Then, the *E_j,_*_norm_ *-F_j_* curve of the whole structure can be plotted to investigate its stressing state characteristics vividly. 

### 2.3. M-K Criterion

Applying Mann-Kendall (M-K) method, which is a generally used trend analysis tool customarily without necessity for samples to conform with certain distributions or interference of a few outliners, the stressing state mutation of the structure can be distinguished through the *E_j,_*_norm_ *-F_j_* curve [31,32]. This paper assumes that the sequence of {*E_j,_*_norm_ (*i*)} (the *i*th load step, *i* is 1, 2, …, *n*) is statistically independent. Then, a new stochastic variable *b_k_* at the *k*th load step is defined as

(4) $$b_{k}=\sum_{i}^{k}h_{i}\left(2\leq k\leq n\right)$$

(5) $$h_{i}=\left\{ \begin{array}{l} +1,\\ 0 \end{array}\right.\begin{array}{l} E_{j,\mathrm{norm}}\left(i\right)>E_{j,\mathrm{norm}}\left(j\right)\\ \mathrm{otherwise} \end{array}\begin{array}{l} \left(1\leq j\leq i\right) \end{array}$$

where *h_i_* is the cumulative number of the samples; “+1” means adding one more to the existing value if the inequality on the right side is satisfied for the *j*th comparison. The mean value *E*(*b_k_*) and variance *HK*(*b_k_*) of *b_k_* are calculated by

(6) $$E\left(b_{k}\right)=k\left(k-1\right)/4\left(2\leq k\leq n\right),$$

(7) $$HK\left(d_{k}\right)=k\left(k-1\right)\left(2k+5\right)/72\left(2\leq k\leq n\right),$$

Under the assumption that the {*E_j,_*_norm_ (*i*)} sequence is statistically independent, a new statistic *BP_k_* is defined by

(8) $$BP_{k}=\left\{ \begin{array}{l} 0\\ \frac{b_{k}-E\left(b_{k}\right)}{\sqrt{HK\left(b_{k}\right)}} \end{array}\right.\begin{array}{l} k=1\\ 2\leq k\leq n \end{array}$$

Then, the *BP_k_* -*F_j_* curve can be plotted. The proceeding of the inverse {*E_j,_*_norm_ (*i*)} sequence is consistent with before, which can form the *TP_k_* -*F_j_* curve. Thereby, the mutation point of the *E_j,_*_norm_ -*F_j_* curve will be determined by the intersection of *BP_k_* and *TP_k_* curves, so that M–K criterion is taken for distinguishing the structural stressing state mutation.

## 3. Experimental Bridge Model

### 3.1. Configuration of the Bridge Model

**Figure 1:**
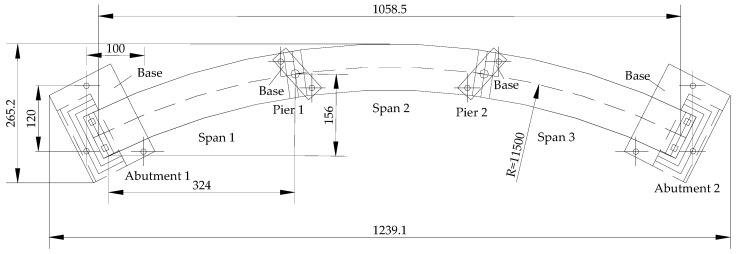
Layout plan of the IACBG bridge model (unit: mm).

**Figure 2:**
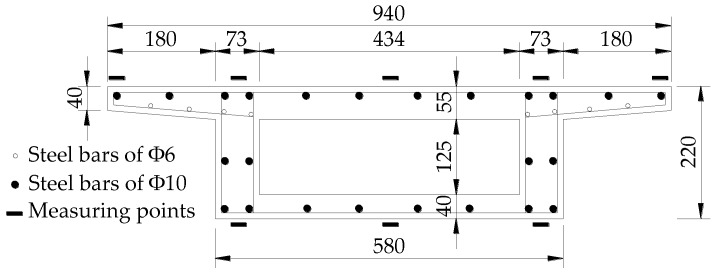
Shape and sizes of the cross section (unit: mm).

According to a 3 m × 25 m curved box girder bridge in Dongguan, Guangdong, China, Lin designed and carried out the experiment of the 1/6.8 scale IACBG bridge model with 3 spans of 3.67 m its shown in Figure 1 [24]. Each abutment is 0.4 m height and 0.2 m thickness and two single rectangular piles with 1.4 m height, 0.1 m × 0.12 m section, and 0.4 m spacing are used for its foundation. Single column pier with diameter of 150 mm and height of 1.56 m is adopted. The abutments are in rigid connection with box girder and piles, and each pier supports the main girder in a single point. The piles, piers and abutments are made of C30 concrete and the upper box girder is C40 concrete. The compressive strength, elastic modulus, and Poisson’s ratio of C30 and C40 concrete are respectively 40.4 MPa and 47.2 MPa, 37.1 GPa and 37.2 GPa, 0.122, and 0.126. The yield strength, ultimate strength, elastic modulus, and elongation of 6 mm and 10 mm steel bars are respectively 416 MPa and 404 MPa, 577.2 MPa and 560.7 MPa, 251 GPa and 169 GPa, and 19% and 22.6%. The layout of steel bars and sizes of the cross section are shown in Figure 2.

### 3.2. Measuring Point Arrangement

**Figure 3:**
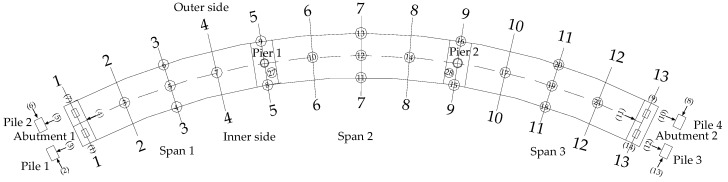
Arrangement plan of testing cross sections and dial indicators.

As shown in Figure 3, displacements and strains of thirteen cross-sections, respectively numbered 1 to 13, are measured in the bridge model. A total of twenty-five vertical displacement dial indicators are arranged on the whole bridge model and three radial displacement dial indicators are respectively arranged on the mid-span webs and the top sections of two piers. Besides, the displacements of the top sections for four piles and the mid-high sections for two abutments are also tested. Symbols ① to ㉘ and (1) to (14), respectively represent the number of each dial indicator on main girder and substructure (piers, piles and abutments). The arrangement of concrete strains on each section for main girder displays in Figure 2. However, due to damaged measuring apparatus, etc., some measuring results really exist large errors. In order to reduce the influence of experimental error propagation on the results of the paper, some measuring results are neglected [33].

### 3.3. Loading Scheme

**Figure 4:**
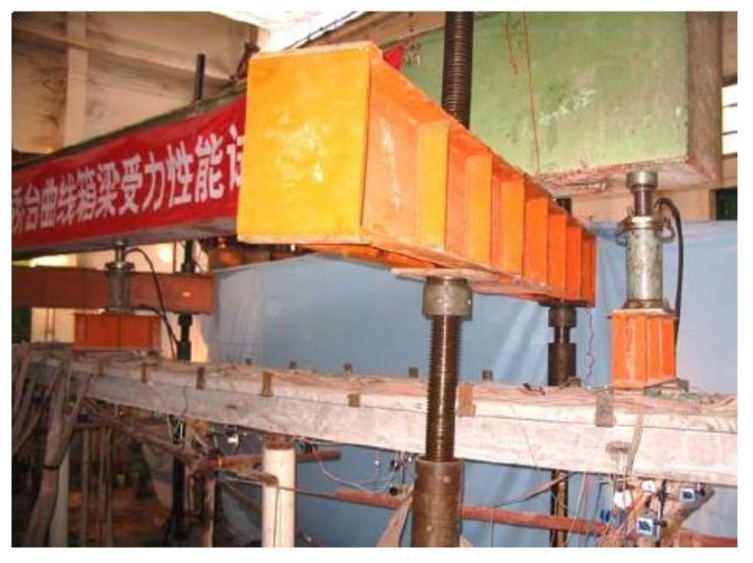
Experimental bridge model and loading apparatus.

A spring-loaded system was used to simulate the action of earth pressure and the horizontal stiffness of soil spring by using the tensile stiffness of steel spring. Each spring element with stiffness of 33.3 kN/m behind abutments and 66.7 kN/m behind piles respectively applied 1 kN and 0.25 kN. Each abutment had ten spring elements and each pile had five ones. The center of side span and the mid-span in longitudinal and both sides of the web in lateral were under step loading shown in Figure 4. Loads were firstly imposed with a fixed increment of 4 kN and then data were collected after holding the load stable for 10 min. When it increased to 20 kN, the increment varies into 2 kN until the bridge model was destroyed. 

## 4. Analysis of the Bridge Model’s Stressing State Characteristics and its Parameters

### 4.1. GSED-Based Stressing State Mode and Characteristic Parameter

Structural stressing state characteristics of the bridge model can be properly manifested by stressing state mode, $S_{E_{j}}={\left[e_{1j}\cdots e_{ij}\cdots e_{nj}\right]}_{j}^{T}$, where *e_ij_* is the GSED value of the *i*th position of the bridge model (sections 1, 3, 5, 7, 9 and 11, abutments 1 and 2, piers 1 and 2, and piles 1, 2, 3 and 4) under the *j*th load. In this paper, the sum of the GSED values is proposed as the parameter to express characterizing structural stressing state mode $S_{E_{j}}$ calculated by

(9) $$E_{j}=\sum_{i=1}^{N}e_{ij},$$

Then the stressing state mode $S_{E_{j}}$ and its characteristic parameter *E_j_* based on the structural stressing state theory would be investigated to reflect the mutation characteristics of the bridge model’s stressing state in the following.

**Figure 5:**
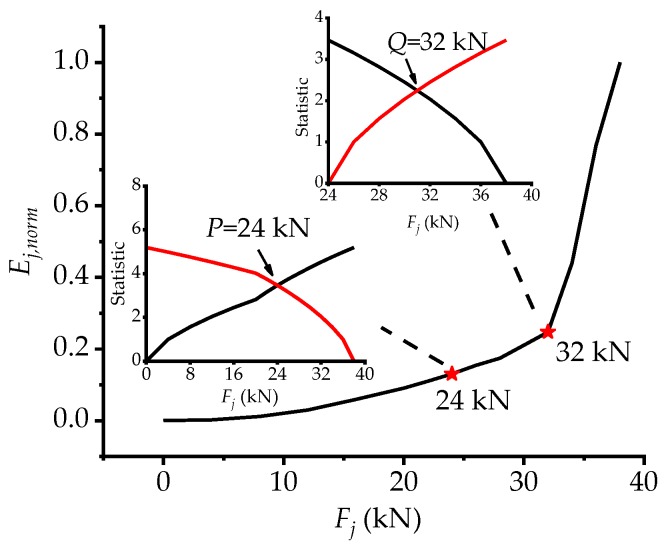
The *E_j,_*_norm_ *-F_j_* and M-K statistic curves.

After normalizing the GSED sum values of the cross sections (section 1, 3, 5, 7, 9 and 11) according to Equation (3), the *E_j,_*_norm_*-F_j_* curve can be plotted to reveal the structural stressing state characteristics of the bridge model in Figure 5 and the two points of the stressing state at loads *P* and *Q* (24 kN and 32 kN) are distinguished by the M-K criterion. Evidently, *E_j,_*_norm_ increases gently before load *P*, displaying stable structural stressing state considered as an elastic working state. Thereafter, the curve still increases relatively stable, indicating that the whole bridge model enters elastic-plastic working state with local plastic development. Due to the intensive development of concrete cracks and the reduction of sectional stiffness, the plastic deformation of the bridge model is large enough to affect the working behavior of the whole structure. After load *Q*, *E_j_*_,norm_ begins to grow rapidly and sharply, so the stressing state of the bridge model enters another stage different from the previous one at all, in spite of not reaching the ultimate load. Hence, load *Q* is defined as the structural failure load, different from the existing failure load, which is defined as the structural ultimate load. It could also be drawn from the explanation above that the *E_j,_*_norm_ *–F_j_* curve can vividly express the structural stressing state characteristics of the bridge model.

### 4.2. Analysis of Stressing State Modes Based on Displacements and Strains

The stressing state modes is formed by structural strains and displacements as follows,

(10) $$S_{\mathrm{DSPL}}=\left[\begin{array}{l} d_{11}\begin{array}{cccc} \cdots & d_{1j} & \cdots & d_{1n} \end{array}\\ \begin{array}{c} \vdots \\ d_{i1}\\ \vdots \\ d_{n1} \end{array}\begin{array}{cccc} \ddots & & & \\ & d_{ij} & & \vdots \\ & & \ddots & \\ & \cdots & & d_{nn} \end{array} \end{array}\right],$$

(11) $$S_{\mathrm{strain}}=\left[\begin{array}{l} s_{11}\begin{array}{cccc} \cdots & s_{1j} & \cdots & s_{1n} \end{array}\\ \begin{array}{c} \vdots \\ s_{i1}\\ \vdots \\ s_{n1} \end{array}\begin{array}{cccc} \ddots & & & \\ & s_{ij} & & \vdots \\ & & \ddots & \\ & \cdots & & s_{nn} \end{array} \end{array}\right],$$

where *d_ij_* and *s_ij_* are, respectively, the displacements and strains on the *i*th position of the bridge model under the *j*th load. Then, they can be plotted as the **S**_DSPL_-*F* and **S**_strain_-*F* curves in the following figures in order to reveal the characteristics of structural working behavior for the bridge model. With regard to the IACBG bridge model, the abutments and the bridge girder are in rigid connection, so the working behavior of piles and abutments would have a certain influence on those of the whole bridge model. 

**Figure 6:**
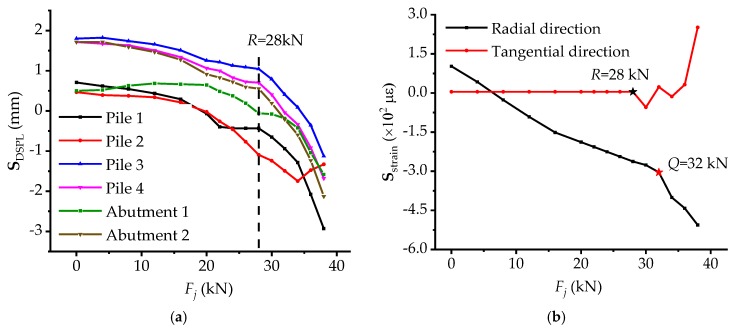
The curves of strain-based and DSPL-based stressing state modes with respect to load for piles and abutment: (**a**) The **S**_DSPL_ -*F* curve for piles and abutment in tangential direction; and (**b**) The **S**_strain_-*F* for pile 2.

As illustrated in Figure 6a, **S**_DSPL_-*F_j_* curves decrease slowly until load *R*, thereafter, except pile 2 nearly all the curves mutate and decrease rapidly, implying that the stressing state of piles and abutments changes from stable one to relatively unstable one. As for pile 2, the change of stressing state embodies in the interior of pile 2 and it is obvious in Figure 6b that the **S**_strain_ of steel bars in tangential direction maintains 0 *με* until load *R*. After that, the curve fluctuates, signifying the relatively unstable stressing state for pile 2. Actually, as for integral abutment bridges, the flexibility of piles, abutments, and piers is usually large, yet there is a large stiffness for girders, resulting in the remarkable and sensitive external features in the loading proceeding, such as deformations, so the tangential displacements of bridge model would tend to mutate in advance. While the real stressing state of the piles, abutments, and piers has not mutated at load *R* coinciding with **S**_strain_-*F_j_* curves in Figure 6b. After load *Q*, the **S**_strain_ in the radial direction mutates and decreases sharply, indicating that the mutation of stressing state for the whole bridge model can embody in the pile as well, and the pile together with the whole bridge model enters failure stressing state stage, indeed. In addition, the large flexibility of the piles, abutments and piers can also bring better self–regulation ability to them, hence, the mutation of displacements at load *R* would have a certain effect on the bridge girder. 

In the bridge model, due to the location where the load is applied, sections 3 and 7 bear sagging moments, while sections 5, 9 and 11 are on the contrary. It is obvious that before load *R* the **S**_strain_ of sections 3 and 7 increase gently and all the measured steel bars are not up to 2392 *με* (yield strain *ε_y_*), meaning that all of them stay in the unyielding condition, which can be regarded as a stable stressing state. While, thereafter, the **S**_strain_ trend of the middle side (MS) and outer side (OS) on section 3 mutates and the steel bars at the position become yielding, indicating that the unstable stressing state of piles would have a certain influence on the upper girder indeed. The phenomenon is owing to self- regulation of the whole bridge model and can be considered as the preparation for the structural stressing state qualitative change (mutation) of the whole bridge. When it comes to load *Q*, there is a sudden and significant increase of the inner side (IS) and OS of section 7, the MS and OS of section 3. In addition, the positions of the yielded steel bars are up to four, respectively the IS and OS of section 7 and the middle side (MS) and OS of section 3, indicating that the stressing state of the bridge model comes out of qualitative change.

**Figure 7:**
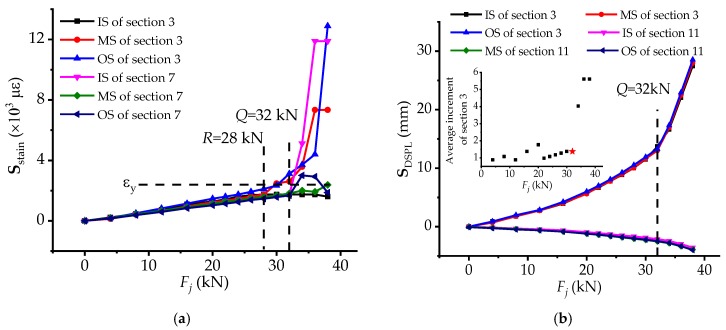
The curves of strain-based and DSPL-based stressing state modes with respect to load for bridge girder: (**a**) The **S**_strain_-*F* curve for sections 3 and 7; and (**b**) The **S**_DSPL_-*F* curve for sections 3 and 11.

As for the **S**_DSPL_ of sections 3 and 11 shown in Figure 7b, the absolute value of displacement in section 3 is evidently much larger than that of section 11, and the **S**_DSPL_ also mutates at the updated failure load *Q*. Finally, **S**_DSPL_ is up to 30mm twice larger than that of load *Q*. Hence, the stressing state of the model can be divided into two stages by the characteristic load *Q* and a great change of structural stressing state has taken place after it, implying that the whole bridge model entering unstable state (failure) stage. 

**Figure 8:**
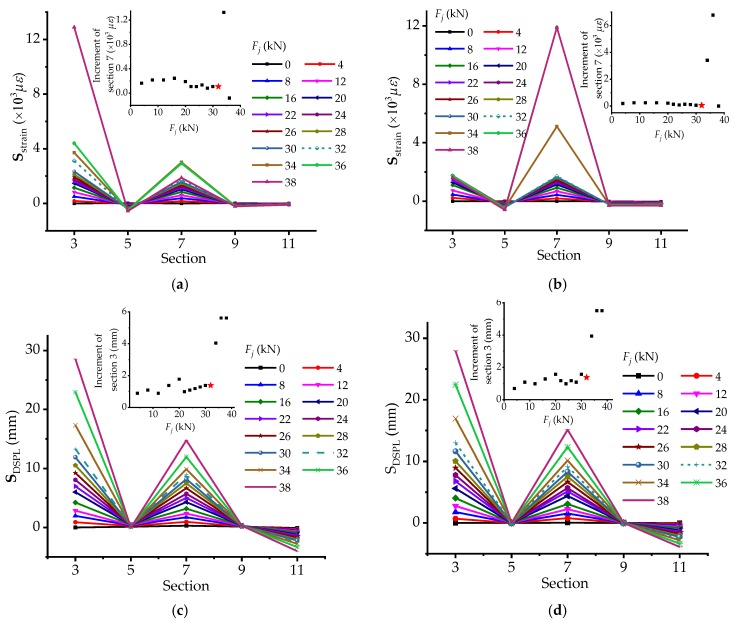
The changing features of strain-based and DSPL-based stressing state modes for cross sections: (**a**) Strain-based stressing state mode for the OS; (**b**) strain-based stressing state mode for the IS; (**c**) DSPL-based stressing state mode for the OS; and (**d**) DSPL-based stressing state mode for the MS.

Easily, the strain-based and DSPL-based stressing state modes **S**_strain_ and **S**_DSPL_ can be plotted in the other form as well. As for the change of the stressing state modes based on strains in Figure 8a,b, it can be seen that the modes mutate a lot at the updated failure load *Q*, in particular for shape and incremental amplitude in Figure 8b. Besides, after load *Q* for the strain steel bars on the OS of the bridge model, section 3 usually plays the main role in the whole sections, while that of the IS one is section 7. In accordance to the curves of **S**_DSPL_ shown in Figure 8c,d, the mutation features of the mode are distinctly observed and the mutated characteristic can exhibit more remarkable at load *Q* in the increment diagram. Hence, the increment of strains and displacements for the critical section could also be used as a parameter characterizing the stressing state mode **S**_strain_, so that more apparent mutations of the stressing state could emerge. 

### 4.3. Component Stressing State Mode

According to the characteristics of curved bridge in complexed stressing state, the experimental bridge model bears different internal forces, including axial force, bending moment and torsional moment, so the component stressing state mode based on stressing state theory can be put forward to investigate the working behavior characteristics of the bridge model under different internal forces. The average sectional displacements of the IS, MS, and OS are calculated as characteristic parameters to express the in-plane bending behavior and the difference value of strain between the OS and IS for out-plane ones under every load step respectively calculated by Equations (12) and (13).

(12) $${\bar{d}}_{i}=\left(d_{\mathrm{OS},i}+d_{\mathrm{MS},i}+d_{\mathrm{IS},i}\right)/3$$

(13) $$\Delta \varepsilon_{i}=\varepsilon_{\mathrm{IS},i}-\varepsilon_{\mathrm{OS},i}$$

where *i* denotes section number (1, 3, 5, 7, 9, 11 and 13); *d*_IS,*i*_ , *d*_MS,*i*_ and *d*_OS,*i*_ are, respectively, the displacement of the *i*th section on the IS, MS, and OS; $\varepsilon_{\mathrm{IS},i}$ and $\varepsilon_{\mathrm{OS},i}$ are, respectively, the strain of the *i*th section on the IS and OS.

**Figure 9:**
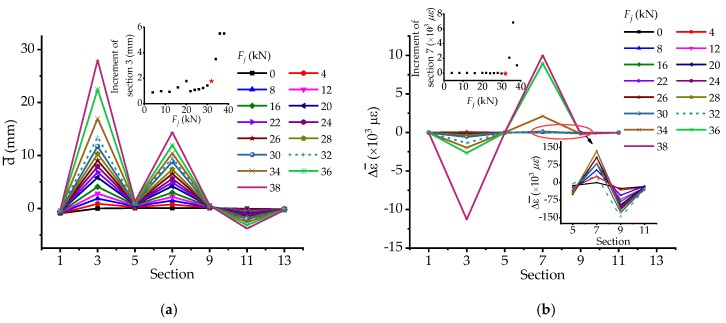
The changing features of generalized bending moment stressing state modes: (**a**) Generalized in-plane bending moment stressing state mode; and (**b**) generalized out-plane bending moment stressing state mode.

It can be seen in Figure 9a that sections 3 and 7 bear sagging bending moments, whereas section 11 bears the reverse one, among them section 3 suffering the largest one, which can be considered as the controlled section under the given load. In addition, when it comes to load *Q*, the average displacement of section 3 increases suddenly and quickly and the peak shape of the curve becomes sharper, displaying twice growth in increment diagram without diminution trend at all. As for out-plane bending moment mode shown in Figure 9b, the out-plane moment on sections 3 and 9 is opposite to that of section 7, different from the condition under the in-plane moment. Besides, before load *Q* each section bears less bending moment thereafter, it multiplies and the stressing state changes a lot as well. Hence, the bending moment including the in-plan and out-plan one could both embody great variation of two structural stressing state stages.

**Figure 10:**
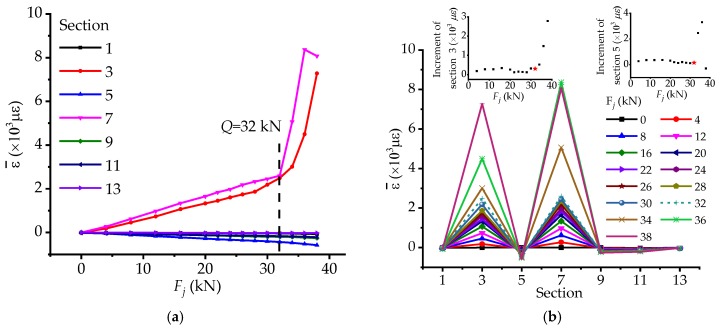
The changing features of generalized axial force stressing state modes: (**a**) The trend changing features of generalized axial force stressing state mode; (**b**) The distribution pattern changing features of generalized axial force stressing state mode.

As for axial force effect, it is proposed to use the average of strain in each section as characteristic parameters to express it and it can be plotted in two ways shown in Figure 10. Both the two figures reflect that the mutation at the load *Q* and the effect to sections 3 and 7 is almost the same, in spite of a little larger for section 7, indicating that axial force has great influence on the two sections.

**Figure 11:**
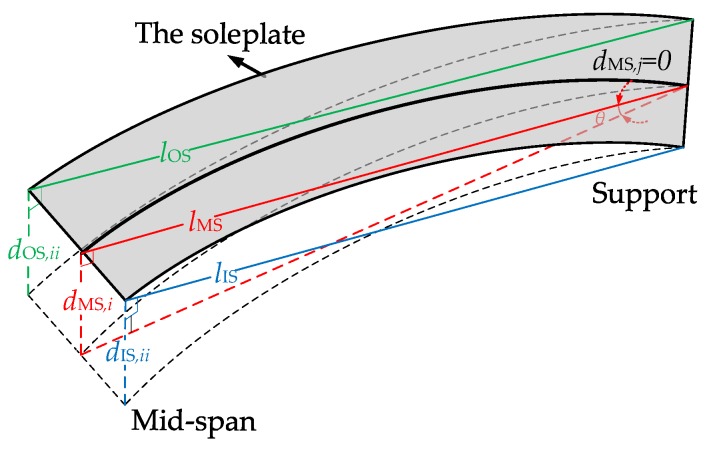
The in-plane bending displacements and rotational angle *θ* for a section.

As for straight girders under vertical loads, the torsional behavior of them can be easily and efficiently presented by the difference values between the displacements of the OS and IS, calculated by Equation (14),

(14) $$\Delta d_{j}=d_{\mathrm{IS},j}-d_{\mathrm{OS},j},$$

where *d*_IS,*j*_ and *d*_OS*,j*_ are, respectively, the displacement of the *j*th section on the IS and OS; *j* denotes section number (5 and 9). While for curve girders the influence of unsymmetrical bending cannot be ignored, which can bring out the difference value of displacement as well. Whereupon the specific construction method of the characteristic parameters, expressing torsional behavior for mid-span section (sections 3, 7 and 11) of every span after eliminating the influence of unsymmetrical bending, is expressed in Figure 11 and, correspondingly, torsional behavior could be calculated by the following Equation (15) to (20),

(15) $$\tan\theta =\frac{d_{\mathrm{MS},i}-d_{\mathrm{MS},j}}{l_{\mathrm{MS}}},$$

(16) $$d_{\mathrm{OS},ii}=l_{\mathrm{OS}}\times \tan\theta ,$$

(17) $$d_{\mathrm{IS},ii}=l_{\mathrm{IS}}\times \tan\theta ,$$

(18) $${\tilde{d}}_{\mathrm{OS},i}=d_{\mathrm{OS},i}-d_{\mathrm{OS},ii},$$

(19) $${\tilde{d}}_{\mathrm{IS},i}=d_{\mathrm{IS},i}-d_{\mathrm{IS},ii},$$

(20) $$\Delta {\tilde{d}}_{i}={\tilde{d}}_{\mathrm{IS},i}-{\tilde{d}}_{\mathrm{OS},i},$$

where *θ* is the angle caused by in-plane bending; *i* denotes section number (3, 7 and 11); *j* denotes section number (5 and 9); *d*_MS,*j*_ is the displacement of the *j*th section on MS; *l*_OS_, *l*_MS,_ and *l*_IS_ are respectively the distance between mid-span and pier section on the IS, MS and OS; *d*_IS,*ii*_ and *d*_OS,*ii*_ are respectively the displacement of the *i*th section caused by bending on the IS and OS; ${\tilde{d}}_{\mathrm{IS},i}$ and ${\tilde{d}}_{\mathrm{OS},i}$ are respectively the displacement of the *i*th section caused by torsion on the IS and OS; $\Delta {\tilde{d}}_{i}$ is the generalized torque on the *i*th section. For other sections (sections 1, 5, 9 and 13), authors still use the Equation (14) to calculate torque shown in Figure 11. 

**Figure 12:**
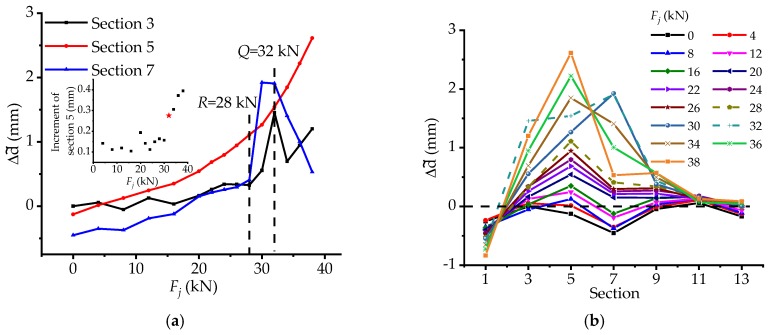
The changing features of generalized torque modes: (**a**) The trend changing features of generalized torque stressing state mode; and (**b**) The distribution pattern changing features of generalized torque stressing state mode.

As is illustrated in Figure 12a, before load *R* the curves of sections 3, 5, and 7 increase slowly, thereafter, those of sections 3 and 7 mutate rapidly, indicating that the torsional stressing state of the bridge is influenced by the interaction among piles, abutments and bridge girder to some extent, urging the change of stressing state for the whole bridge model. After load *Q*, the curves of sections 3 and 7 decrease quickly and that of section 5 increases promptly. While, from the incremental diagram, it seems that the mutation of the curve for section 5 starts at 30 kN, in fact the phenomenon is caused by the effect of its near sections. At that time section 3 and 7 vastly twist to the IS bringing extra torsional deformation of section 5 based on the growth of itself. Even if the great increment of section 5 produces at load *Q*, the increment of 34 kN still increases a lot exactly signifying the mutation of the stressing state after load *Q*.

Moreover, it can be seen that at the beginning in Figure 12b, the cross sections near abutments (sections 1 and 13) and the midspan of 2-span (section 7) twist from outside to inside, while the others twist oppositely. After load *R*, the torque of section 7 changes suddenly and the peak of the curves varies from sections 5 to 7 differently from the previous at all. Although the interaction among piles, abutments, and bridge girders causes the stressing state change of the bridge model, due to the self-regulation ability of the bridge model, the bridge model still tries to keep the previous state. Then, when it comes to load *Q*, the torque effect of section 3 increases sharply, up to 50% rate growth, even larger than that of 38 kN. Thereafter, the distribution pattern of the stressing state mode turns into that before load *R* in shape, which signifies the changing of the stressing state, and the value of section 5 continues to increase faster than before. Besides, in view of the whole processing of load, section 5 plays a major control role for torque almost all the way compared with other sections. 

So through furthering analysis the changing features of component stressing state modes, it could be stated that the modes formed by the characteristic parameters of different internal forces also mutate at the updated failure load. And the M-K failure criterion could effectively distinguish different structural stressing states indeed. Besides, the analysis could also embody that the different internal forces play different roles in loading process.

### 4.4. Coordinate Working Features of the Bridge Model

Here, a research is tried to see whether the structural coordinate working behavior could be reflected in a sense of structural stressing state or not. From this consideration, the correlation curves about internal forces and internal response to external import work are plotted in order to investigate some unseen coordinate working features.

**Figure 13:**
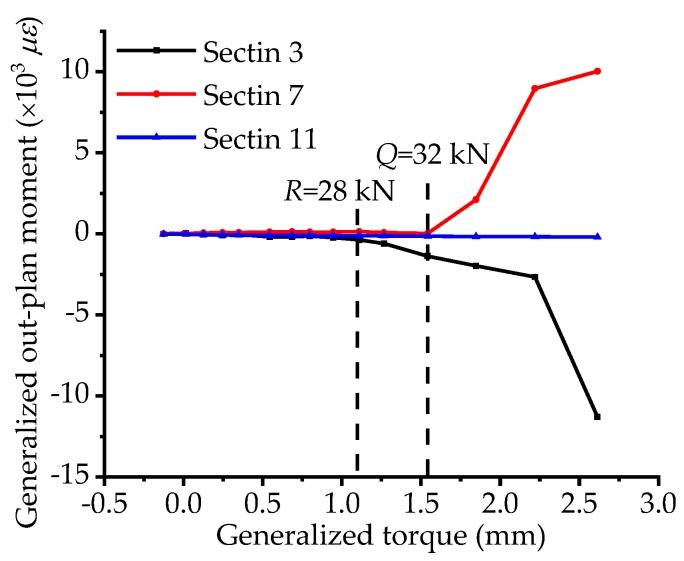
The correlation between torque and out-plan bending moment.

For the sake of furthering study the curvature effect on the generalized torsional and out-pane bending behavior, the correlation curves between the generalized torque and out-plane bending moment are plotted for sections 3, 7, and 11, shown in Figure 13. Before load *R*, the curves of sections 3, 7, and 11 nearly maintain straight, indicating that the degree of coupling for the two internal forces is relatively low. Then the curve of section 3 begins to increase slightly and the stressing state of the whole bridge model starts to vary. Besides, the mutation feature is also clearly observed at load *Q*, and this could also be indicated that the updated failure load is an essential attribute of structural working behavior and an objective law complying with the natural law from quantitative change to qualitative change of a system, indeed. Meanwhile, the characteristic parameter for generalized torque expressed by the sectional displacements could be considered as an external presentation of torsional stressing state, and that for generalized out-plan moment expressed by the strains could be considered as the internal presentation of bending stressing state. The qualitative variation of sectional stressing state at load *Q* is significantly embodied in the internal presentation of out-plan bending behavior.

**Figure 14:**
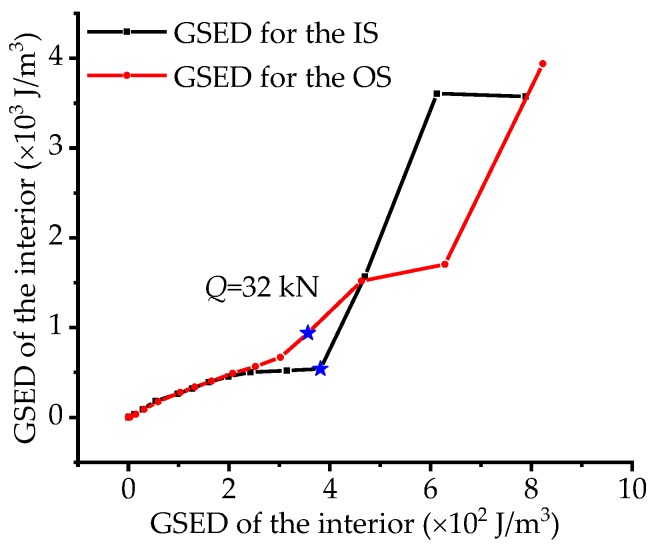
The correlation of the GSED between the exterior and interior.

In order to study what kind of internal response to external import work for the IACBG bridge model, the correlation of GSED between exterior and interior for the IS and OS is calculated by Equation (21) to (24),

(21) $$E_{k}=\sum E_{k,i},$$

(22) $$E_{k,i}=\frac{1}{2}E\varepsilon_{k,i}^{2},$$

(23) $$E_{r}=\sum E_{r,j},$$

(24) $$E_{r,j}=\sum F^{j}d_{r,j},$$

where *k* is 1, 2, respectively, on behalf of the IS and OS; *E_k_* is the GSED of interior on the IS or OS; *E_k,i_* is the GSED of interior for the *i*th section; *i* denotes section number (3, 5, 7, 9 and 11); *ε_k,i_* is the strain of the IS for the *i*th section; *r* is 1 and 2 respectively on behalf of the IS and OS; *E_r_* is the GSED of exterior on the IS or OS. *j* denotes section number (3 and 5); *E_r,j_* is the GSED of exterior for the *j*th section; *F^j^* is the load force of the *j*th section on the IS or OS; *d_r,j_* is the displacement of the *j*th section on the IS or OS. As is illustrated in Figure 14, before load *Q* the curves slowly increase and nearly overlap each other, indicating that the response of the IS and OS is in accordance with each other and the whole bridge model stays in stable stressing state stage. Then the curves begin to fluctuate crisscross, implying that the response of the bridge model change into instability. Besides, the curve for the IS increases suddenly and rapidly after load *Q*, which also consists with the previous description.

As expounded above about the correlation of internal force and GSED, it could not only help us understand the internal relationship and the effect of curvature on the torsional and out-plane bending behavior, but also the response features of the curved bridge model in the whole loading proceeding. What’s more, they could contribute to the rationality of the updated failure load defined at load Q, indeed, and help investigate the changing features of the coordinated work ability through correlation contrast.

## 5. Conclusions

The stressing state theory is applied into an investigation of the working behavior for an integral abutment curved box-girder bridge model, revealing its stressing state’s qualitative mutation characteristic at a certain load and unseen working behavior features in the loading process. The following conclusions are derived from this research:

1. The normalized GSED values can effectively model structural stressing state and reflect their working behavior features. The M-K criterion is applied to detect failure load *Q* through *E_j_*_,norm_-*F_j_* curve, hence two stressing state stages of the bridge model are separated in its whole loading process. Then the effectivity and rationality of the M-K criterion are verified through the analyses of strain-based and DSPL-based stressing state modes, component stressing state modes and correlation features of characteristic parameters.
2. The concept of component stressing state modes is proposed based on the inherent in-plane and out-plane bending and torsional behavior of the bridge model, which is modeled by the average displacement, strain difference, and displacement difference. And the working behavior of the bridge model under different internal forces and their roles in the structural failure process could be reflected as well.
3. The concept of structural coordination working behavior is proposed based on the coupling relationship of different internal forces and structural response under load, which modeled by the generalized torsional and out-pane bending behavior and the GSED of exterior and interior. Coordinate curves diverted from this concept can characterize the structural coordination working behavior features and reflect qualitative mutation characteristics as well.

In a word, the new knowledge of the bridge model’s working behavior revealed by the structural stressing state theories provides a new reference to the structural design and a new way to structural analysis.
